# Assessing the role of actors in river restoration: A network perspective

**DOI:** 10.1371/journal.pone.0297745

**Published:** 2024-04-16

**Authors:** Gabriela Ioana-Toroimac, Cătălina Stoica, Gabriela Adina Moroșanu, Ionuț Andrei Șandor, Dana Maria Constantin

**Affiliations:** 1 Faculty of Geography, University of Bucharest, Bucharest, Romania; 2 National Research and Development Institute for Industrial Ecology–ECOIND, Bucharest, Romania; 3 Institute of Geography, Romanian Academy, Bucharest, Romania; Ovidius University of Constanta: Universitatea Ovidius din Constanta, ROMANIA

## Abstract

The aim of this study was to identify and characterize the actors involved in river restoration in Romania by untangling the complex networks that they are part of. Actors were considered nodes in a social network, tied by a common action or project. The nodes were characterized through the centrality metrics of a network. The network of actors in river restoration in Romania has a low density and high average path length and diameter for such a spare network, which suggests that knowledge transfer and collaboration are difficult. In the context of a highly centralized water governance system in Romania, the National Authority for Water exerts power and influence in river restoration as well. It has numerous partners, both disconnected and well connected in the network, therefore, it tends to be connected to everybody, as well as gets and diffuses knowledge. Yet, other actors are willing to get involved in river restoration in Romania. NGOs and research organizations have a central position in the network and play various roles (e.g., unconcerned influencers, gate-keepers, pulse-takers). As a recommendation for governing river restoration in Romania, we urge the creation of a national programme focused on river restoration, where the call for projects would be coordinated by the National Authority for Water and open to its territorial subordinates in agreement with stakeholders from various sectors and domains.

## Introduction

After decades of hard river engineering carried out for flood defense and water resources supply, since 2000, restoring a river’s hydromorphological conditions has become a major topic in the European Union (EU). Hydromorphological conditions refer to continuity, connectivity and water flow. River continuity is the key to achieving a good status for European waters according to the Water Framework Directive (WFD). The EU’s biodiversity strategy also aims to restore at least 25,000 km of free-flowing rivers by 2030 [1] and thus to meet the objectives of the Decade on Ecosystem Restoration [2]. Restoring longitudinal continuity of rivers promotes the passage and migrations of fish species [3] and sediment movement [4]. Restoring lateral hydraulic connectivity between wetlands, fringe habitats and riparian lands and the adjacent river channel is extremely important to maintaining natural functioning of floodplain wetlands [5]. In brief, river restoration groups various activities under the same umbrella with the aim of improving the ecological quality of rivers [6]. In the context of the WFD, river restoration and all other actions should be implemented by “competent authorities” (normally state agencies) together with relevant stakeholders that need to be informed, consulted and actively involved [7] under various forms of ‘hybrid’, ‘polycentric’ or ‘multilevel’ governance models [8].

Therefore, river restoration is as much about nature as it is about navigating through actors, political agendas and tenure arrangements [9]. Different restoration trends may be explained by the different types of stakeholders and professions involved [6]. River restoration appears to be a complicated issue in terms of the ecology of games, namely decisions made by networks of actors (e.g., authorities, non-governmental organizations, companies, research bodies) interacting in multiple forums at different geographic scales, addressing a myriad of interconnected issues [10]. The joining together of multiple actors can have several implications, such as power dynamic [11], redistribution of power across actors, different barriers and bridges that might impede or foster coordination [12], or even conflicts [13, 14]. As a particularity, it is even more difficult to govern fluvial systems with natural boundaries different from political ones [15]. As an example, floodplain restoration is driven by flood management [5] with a permanent quest for equilibrium between engineering works for defense and coping with hydrological extremes through nature-based solutions. Thus, restoration projects must be able to balance various conflicting needs and interests [5, 16]. The way that the public and private actors collaborate to achieve consensus on the planning and implementation of a project ensures the success of river restoration [17]. Therefore, it is necessary to analyze and reveal the functions, structures, outcomes, and governance potential [12] in the area of river restoration.

Relationships between actors conducting river restoration can be understood as a network of collaborators [18]. Actors impact a policy issue depending on their position in the network and on their own particular interests. The degree to which actors are connected to each other and their level of involvement can determine their degree of influence. Therefore, individual actors can form a bridge between other various stakeholders and are trusted and heard in several parts of the network [18]. The position of an actor in the ecology of games in water governance can be quantified by using social network analysis [19]. Social network analysis can help identify structures and patterns of interactions between actors and highlight examples of good practices. Thus, it can play a critical role in improving water and environmental governance by analyzing the present status of the role of certain actors and their relations and simulate the impact of further collaborations [20–24].

The aim of the paper is to identify and characterize the actors involved in river restoration through untangling the complex networks that they are part of. Our case study is at the country-scale of Romania, because of the unitary policy implemented at this level. We (i) identify actors in selected river restoration solutions, (ii) design a network of actors involved in these solutions, and (iii) further analyze the functional role of these actors in the network. Finally, we comment on the position of actors in the network in terms of expectations and recommendations. Our findings provide knowledge for decision makers that help to find ways to improve the communication inside this network of actors from our case study.

## Case study

Due to both climatic conditions and strategy drawbacks, Romania is severely impacted by riverine floods, which are the most frequent natural hazards in the country and also most severe in terms of economic assets according to EMDAT database [25]. Romania has inherited a centralized governance from the communist regime (1947–1989), manifested through one institution taking the key decisions. Later, in 2007, Romania became an EU Member State. Since 2007, Romania has implemented the EU water policy (Water Framework Directive 2000/60/CE), as well as the other European regulations. The Romanian National Authority for Water and the Ministry of Environment, Waters and Forests are the designated authorities for implementing the EU water policy in Romania. Recently, Rowbottom and collaborators [26] underlined that legacy generates sticking points in implementing multi-level governance and that the water governance in Romania is still a highly centralized system, with transposition of EU directives at the national level, feeding directly into statutory legislation at lower level.

Romania is part of the Danube River Basin, which covers almost entirely its territory. Furthermore, Romania occupies approximately one third of the Danube watershed. Romania is integrated into the international Danube River District, including 11 administrative sub-basins governed by 11 river basin agencies. Consequently, Romanian territory benefits from 11 River Basin Management Plans (RBMPs) elaborated unitarily for each sub-basin, indicating that a centralized water governance system was responsible for their accomplishment [27]. The RBMPs provide, among others, a ‘programme of measures’ aiming to restore a river’s hydromorphology towards a good ecological status. However, out of the total number of measures, only 8.7% concern hydromorphological and natural water retention measures, while the rest concern other types of measures (60.6%), e.g., against pollution (25.2%), to protect biodiversity (2.6%), and to fight climate change (2.6%) [28]. Within, the ‘programme of measures’, the hydromorphological and natural water retention measures received 2.3% of all funds while actions to fight pollution received 86% of all funds [29]. Additionally, other river restoration solutions in Romania have remained on paper mostly due to financial reasons [30]. Romania is the next to last country among EU Member States when it comes to prioritizing hydromorphological and natural water retention measures at a national scale (based on the quantification of measures published by [28]). Therefore, we hypothesize that Romania has less experience in river restoration when compared to other EU Member States.

## Data and methods

### Planned and completed solutions for river restoration and the actors involved

In this paper, “river restoration” was defined as a physical measure that modifies the physical habitat (i.e., hydromorphological conditions). Here, “river restoration” is not defined by the goal. Various goals may require interventions on the hydromorphological conditions.

To create a database, firstly, we did an extensive search for river restoration actions or projects in Romania. The list of actions and projects corresponds to the sources we used and to the time interval of our analysis. The list is open and can be updated with all new planned actions and projects that receive funds for river restoration.

- An action was considered a technical solution or study planned to improve the conditions of a river reach by a physical measure that modifies the hydromorphology. First, we extracted actions from the RBMPs (2016–2021). Then, we searched for similar actions in other documents named in these RBMPs. We found numerous actions (i.e., nature-based solution for flood risk mitigation) that can be considered relevant for river restoration in the programme of measures of Flood Risk Management Plans (2016–2021).
- A project was defined as a set of completed (or ongoing) actions that modified a river reach’s hydromorphological conditions to improve the ecological conditions and status. We searched for independent river restoration projects implemented in Romania, mostly at reach-scale. Firstly, we used the archived website of River restoration EU LIFE project containing projects with various funding sources according to [31]. Then, we updated the list with information from the LIFE Programme database [32] and from the Operational Programme for Big Infrastructure of the Romanian Ministry of Environment [33]. The found projects cover the 1994–2020 time interval.

Secondly, we identified the actors involved in all these actions (responsible actors) or projects (partner actors), and we quantified the number of measures or projects in which each actor was involved. We labeled actors based on the scale they operate on (i.e., local, regional/county/river basin, national, international) and according to their main sector of activity: public authorities (national, regional, local authorities), NGOs, research organizations (universities, research institutes, consultancy), and private companies (industry).

### Social network centrality

With the list of actors *per* action and project, we created the network of actors involved in river restoration solutions. In this network, an actor is considered to be a node and is connected through an edge to other actors if they participate in the same action or project. An edge is a common action or project between two actors. Our network is undirected [34], with bidirectional collaboration between project partners (i.e., the actors can exchange information in both ways). Using this undirected network of actors, we computed network level indicators (i.e., number of edges, density, average path length, diameter) and node level indicators (i.e., degree, betweenness, eigenvector). Network-level indicators give an overall picture of the interconnectedness between actors. Node-level indicators characterize the position and indirectly the role of each actor in the network. Table 1 contains the definition of these indicators and their significance in the context of our analysis of actors involved in planned actions or completed projects in Romania. All analyses for social network centrality were conducted with NodeXL [35]; all network representations were drawn with Gephi [36].

**Table 1:** Definition and significance of network metrics used in this study.

Measure	Definition	Significance in this study
Network-level metrics		
**Number of edges**	For undirected networks, the number of edges *E* is the number of unique nonzero elements in the adjacency matrix *A_ij_* [37]. $E=\frac{1}{2}\sum_{ij}A_{ij}$	This metric is equal to the number of actions/projects connecting at least two actors. Edges with duplicates mean that two actors are in several common projects.
**Density**	The density is the number of edges *E* in the observed network relative to the total number of possible edges in a completely connected network (where N is the total number of possible connections). For the adjacency matrix *A_ij_*, the density *k* is given by: $k=\frac{2E}{N(N-1)}$ When *k* is equal to 1, the network is fully connected. Otherwise, the value of *k* indicates the proportion of all possible connections that are actual connections [34, 37].	The density of the network shows how well the actors are connected between them. A high density shows that there are common actions/projects between every two actors. In networks where connections are informational, a high density positively facilitates the dispersion of information [23]. Sparsely connected networks show a power-law distribution in which most sites have only few connections [38].
**Average path length**	It is the average number of edges that must be traversed to connect any two nodes in the network.	A path is a route across the network that traverses node to node along the edges of the network. The average path length measures the efficiency of information diffusion in the network. A long average path length means that actors are located far from each other and they need time to communicate.
**Diameter**	It is the longest distance/path length between the two most distant connected nodes in the network or the longest of all the calculated path lengths [34].	Densely connected networks have a short average path length and short diameter [34, 38].
**Node-level metrics**		
**Degree centrality**	The degree is the number of connections the node has with other nodes. The degree *k_i_* of the node *i* in an undirected network is the number of connections the node *i* has with other *j* = 1…n − 1 nodes from the adjacency matrix *A_ij_* [37]. $k_{i}=\sum_{j\neq i}A_{ij}$	The high degree centrality denotes actors linked with many participants, who are likely to hold most information or who can quickly connect with the wider network. These actors can be considered as hubs [23].
**Betweenness centrality**	The betweenness centrality *C*_*B*_ is the frequency with which a node is located on the shortest path between any pairs of nodes, accounting that the connection between nodes might be stronger along paths with more intermediate nodes that are strongly connected than paths with fewer weakly-connected links. $C_{B}(i)=\frac{1}{\left(N-1)(N-2\right)}\sum_{i\neq j,i\neq k,j\neq k}\frac{g_{jk}(i)}{g_{jk}}$ where *g_jk_* is the number of shortest paths connecting *j–k* and *g_jk_*(*i*) is the number that actor *i* is on; N is the number of node pairs in the network that does not include node *i* [37].	In other words, it is a measure of the tendency of connecting nodes which would be otherwise disconnected. An actor with a high betweenness may play an important role in the network because they can control the flow of information and facilitate the communication between actors otherwise disconnected in the network, namely brokers [23].
**Eigenvector centrality**	The eigenvector centrality *C*_*E*_ is an indirect measure of centrality determined by the centrality scores of the nodes to which the node of interest is connected. The eigenvector is a degree-based centrality which measure the global prominence of a node in the network. Represents the sum of the eigenvectors of the sites that the site of interest is connected to: $C_{E}=x_{i}=\frac{1}{\lambda_{1}}\sum_{j=1}^{N}A_{ij}x_{i}$ where λ_1_ represent the largest eigenvector of matrix A_ij_ and *x*_*i*_ is the eigenvector of the node *i* [33].	A high eigenvector score means that a node is connected to many nodes who themselves have high scores and are well connected [39].

We computed three networks that we analyzed through social network analysis. 1) The sub-network of planned actions consists of actors responsible for implementing measures as stated in the documents that we used. For example, a River Basin Agency, with the approval of the National Authority for Water and of the Ministry of Environment, Waters, and Forests may complete a nature-based solution in the river floodplain such as reconnecting an old meander. In the above-mentioned example, each actor is tied through an edge to the other two actors. 2) The sub-network of completed projects regroups actors that join in for a proposal in a funding competition. For example, a LIFE project related to modifying the hydromorphology (by reducing erosion and increasing longitudinal and lateral connectivity) in order to improve certain components of the ecological status has several beneficiaries (e.g., Environmental Protection Agency Gorj, Invisible Nature Consultancy, University of Bucharest) as stated in the general database of the programme. In the previous example, each beneficiary is an actor with two connections. 3) Hypothesizing that the sub-network of planned actions and the sub-network of completed projects share common actors, the overall network comprises the two previous sub-networks. The data used to compute the networks are found in S1 Table.

### Holistic stakeholder mapping

We adapted the 2D form of the holistic model of stakeholder mapping developed by Sedereviciute & Valentini [40] to our network of actors. This 2D model integrates the social network analysis in the Stakeholder Salience Model by Mitchell, Agle & Wood [41]. One dimension of the 2D model measures the way in which power is distributed across the network, equal here to the degree centrality. The other dimension of the 2D model is the degree of interest, translated here by the number of measures or projects in which an actor is involved out of the total number of solutions per country. An actor with a high number of measures or projects expresses an interest in river restoration and possesses the legitimacy to participate in decision making. The 2D holistic model aims to classify actors in four categories: *concerned influencers* (high interest, high power), *concerned lurkers* (high interest, low power) that should be kept informed, *unconcerned influencers* (low interest, high power) that should be kept satisfied, and *unconcerned lurkers* (low interest, low power). Actors with high power tend to work with everyone.

We also classified the actors depending on their relationship between eigenvector centrality and betweenness centrality. This analytic approach was previously used by Motta and collaborators [42] based on the idea of Valente and collaborators [43] of correlating the network centrality metrics. Three categories of actors can be identified: *gate-keepers* with the ability to bridge between the functional basis of the network and the wider community of nodes (high betweenness centrality and low eigenvector centrality values); *pulse-takers* with easy access to other central nodes as well as to the rest of the network (high eigenvector centrality and low betweenness centrality values); and *dual functionality* that are seeking and disseminating information (high eigenvector centrality and betweenness centrality values).

The statistical correlation in the two analyses (degree versus number of projects and eigenvector versus betweenness) was verified through the Spearman correlation test.

## Results

### Overview of solutions and actors

**Fig 1:**
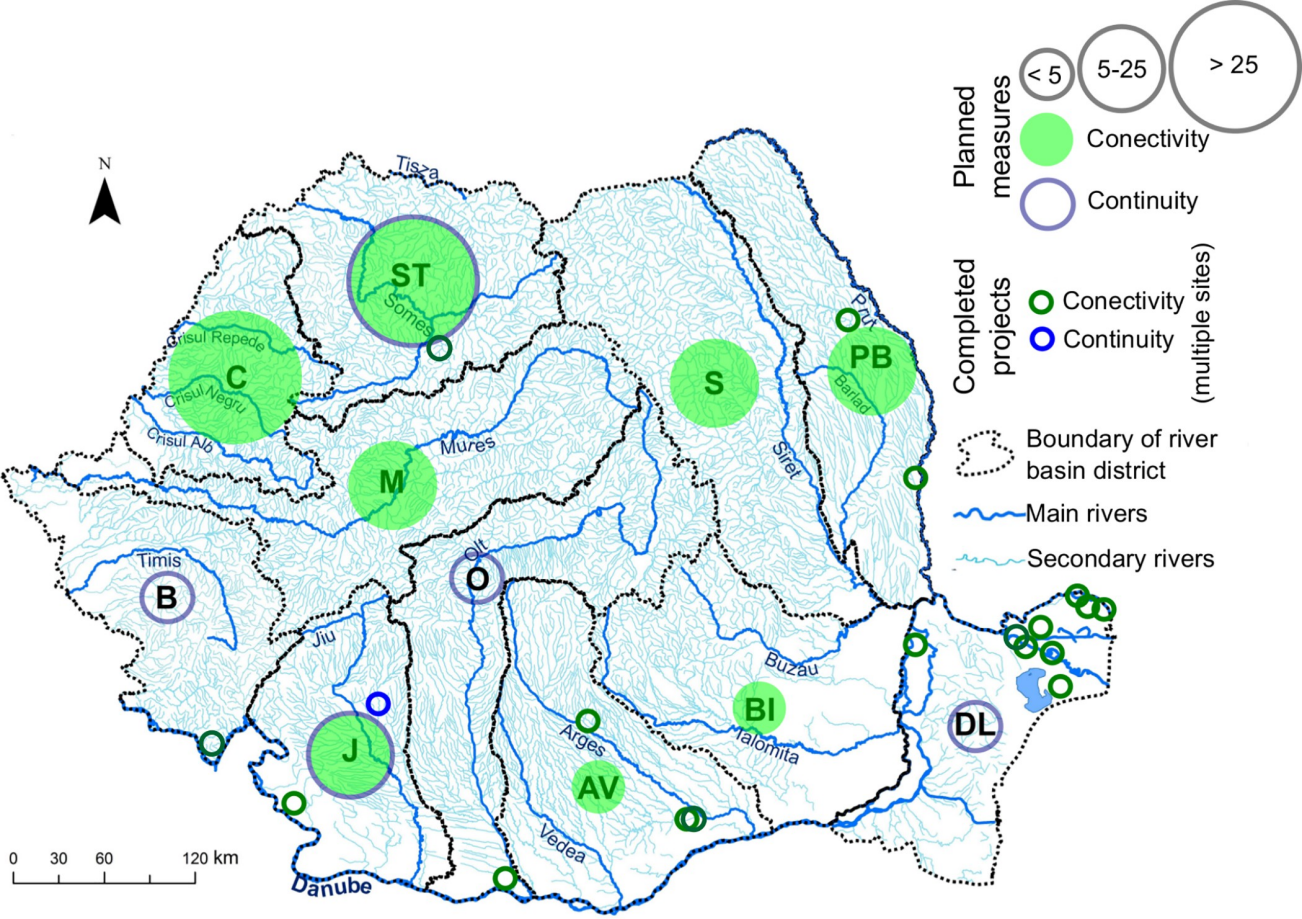
Spatial distribution of solutions for river restoration in Romania. (The number for the planned measures was placed in the middle of the river basin; size of the circle indicates the number of solutions); acronyms of river basins or hydrographic districts: ST = Someș-Tisa; C = Crișuri; M = Mureș; B = Banat; J = Jiu; O = Olt; AV = Argeș-Vedea; BI-Buzău-Ialomița; S = Siret; PB = Prut-Bârlad; DL = Dobrogea-Litoral; the dataset of rivers and boundaries was extracted from WISE WFD Reference Spatial Datasets [44].

**Fig 2:**
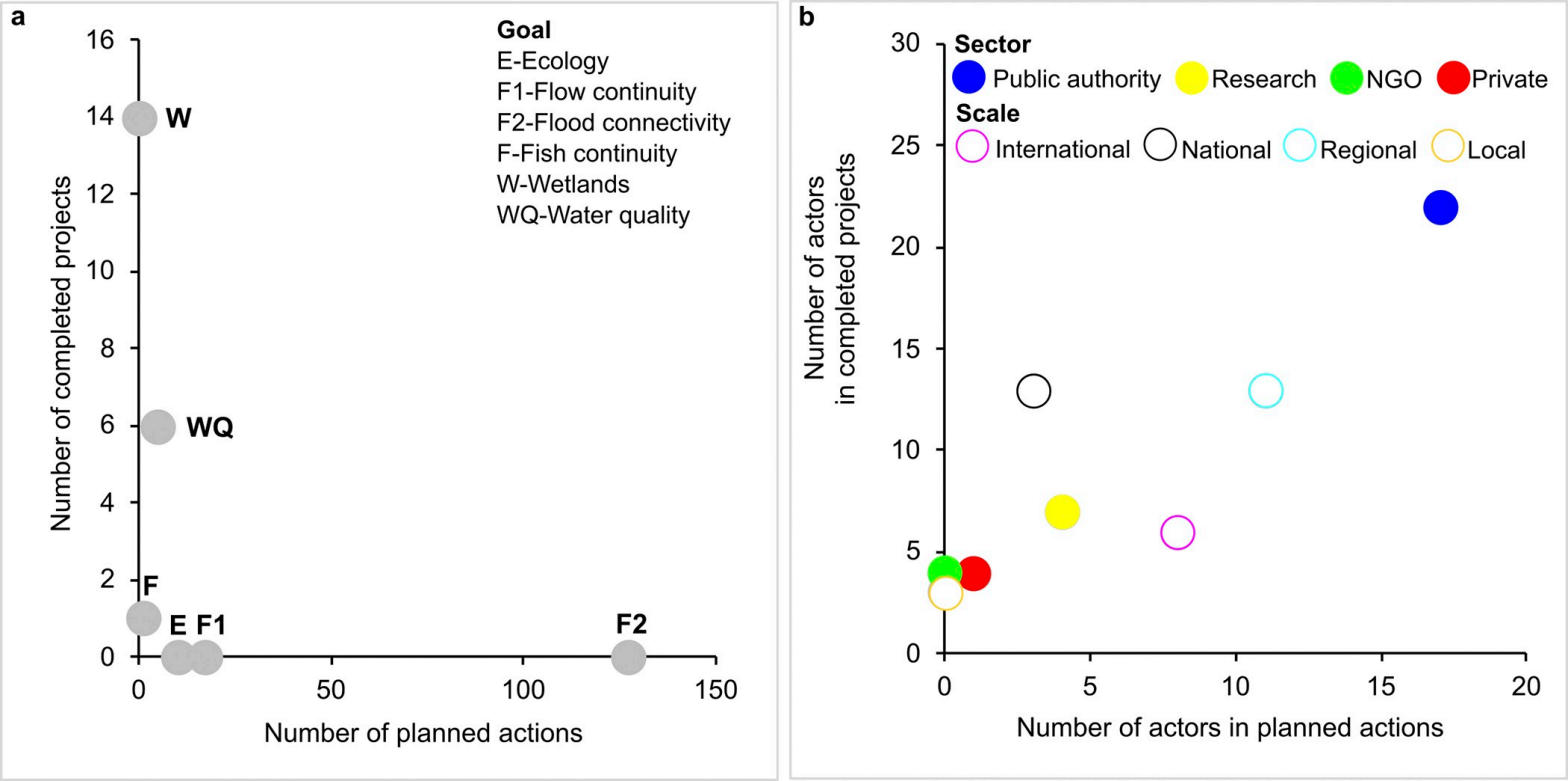
Overview of solutions and actors in river restoration in Romania: a) main goal; b) type of actor.

We found 180 river restoration solutions in Romania, out of which 161 represent planned actions and 19 are completed projects. Concerning the geographical position of all solutions, 30 planned actions or completed projects are found along the Danube River and its delta. The others are unevenly distributed across the country along the Danube’s tributaries (Fig 1). All completed projects are located in protected areas (i.e., in 1 UNESCO, 4 Ramsar, and 4 Natura 2000 sites). Concerning the goal of these solutions, 127 aim at restoring floods in the floodplain (i.e., nature water retention measures), 17 deal with restoring flow continuity, 14 seek to create wetlands, 11 aim for improving water quality, 2 are concerned with improving fish continuity, and 9 have other ecological purposes (Fig 2A). All restoration actions for mitigating floods’ effects and restoring flow conditions are in the planning phase at the moment. All restoration actions aiming to creating wetlands and improving water quality are already completed.

We counted 57 actors involved in river restoration in Romania (S1 and S2 Tables). Most of the actors are public authorities (38 out of which 19 for water, 9 for environment, 5 for forestry, and 5 for public administration), while the rest are from the research sector (11 actors), private companies (4 actors), or NGOs (4 actors). These actors operate at regional scale (24 actors), international scale (14 actors), national scale (15 actors), while the local scale is poorly represented (only 4 actors). Fig 2B regroups the actors in planned actions and completed projects. Public authorities are well represented in both categories of solutions. Both NGOs and local actors are present only in completed projects. Overall, completed projects are characterized by a larger number and variety of actors.

**Fig 3:**
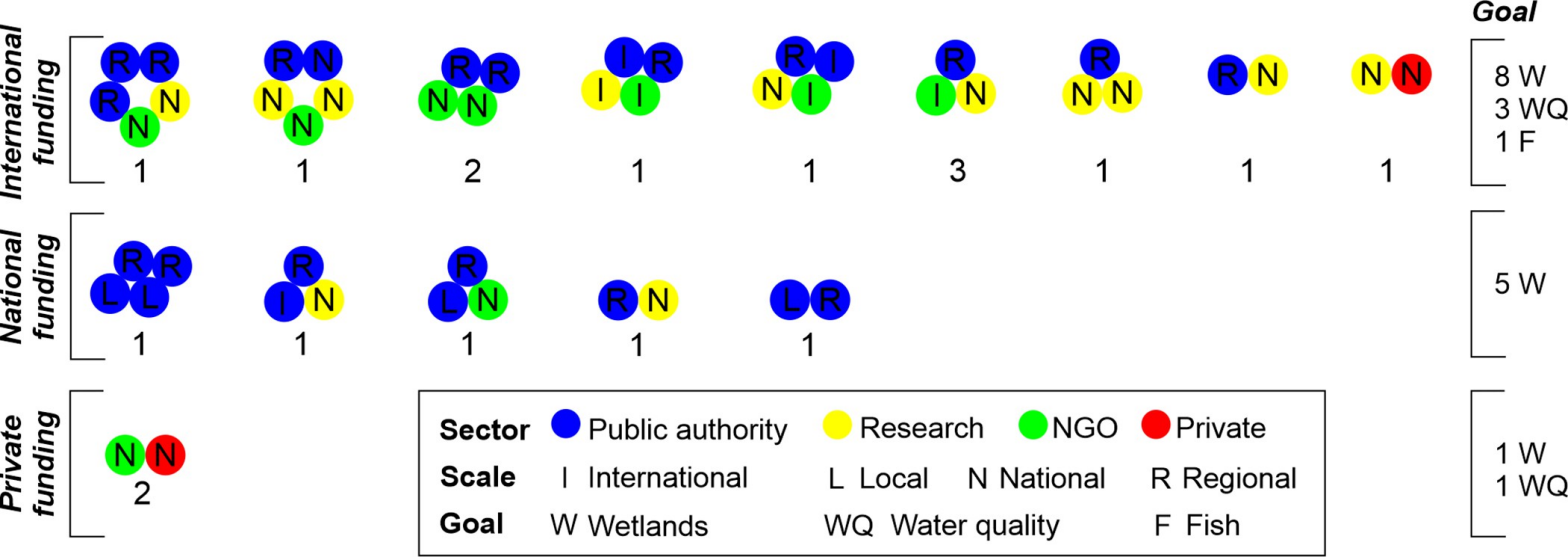
Synthesis on actors in river restoration completed projects in Romania.

Concerning the completion of river restoration projects, we found a variety of partnerships (Fig 3). The following recipes succeeded: 12 out of 19 projects were the result of the collaboration between public authorities and researchers with or without other partners; the majority of the international partners work with public authorities and research partners. International versus local partners, public authorities versus private companies were never found in the same project.

Concerning the funding of these 19 completed projects: 12 were financed at international level (7 by EU LIFE Programme, 3 by Global Environment Facility, and 2 by the Netherlands Government), 5 by the Romanian Government, and 2 by private companies. The majority of international partners came with funds. On the contrary, local partners were financed only by the Romanian Government. Private companies financed their own projects. Concerning the relation between the goal of the project, the actors involved and the funds available, we found that international funding focused on a larger variety of goals, while national funding focused on wetlands creation.

### Network of actors involved in river restoration

**Fig 4:**
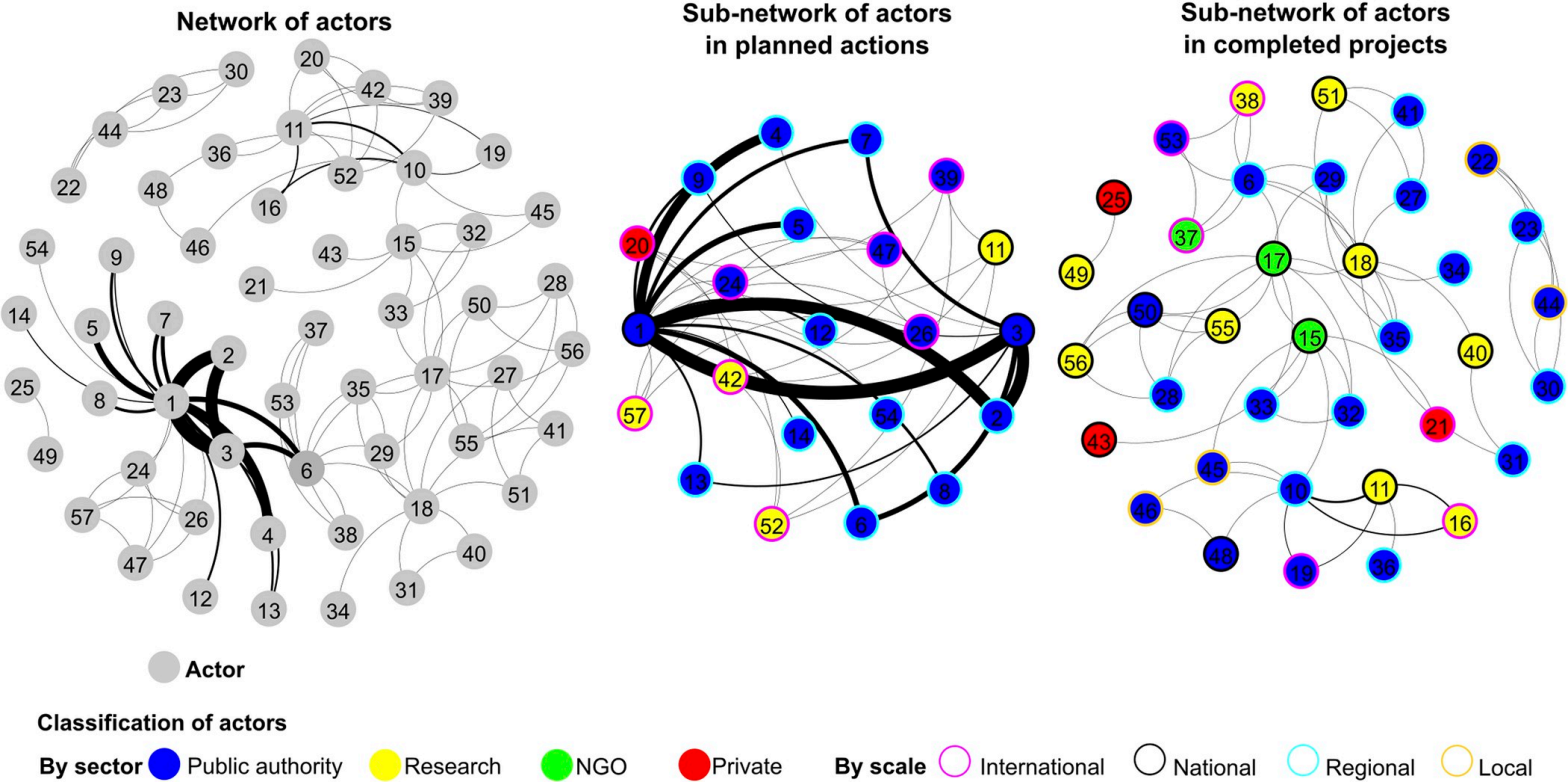
Network of actors in river restoration in Romania (Fruchterman-Reingold layout)–overall network (left), sub-network of planned actions (middle), sub-network of completed projects (right): all nodes are represented equally; edges thickness is comparable in all networks; numbers of actors: 1 –National Authority of Waters; 3 –Ministry of Environment, Waters and Forests; 6 –River Basin Authority Prut-Bârlad; 10 –Danube Delta Biosphere Reserve Authority; 11 –Danube Delta National Institution for Research and Development; 15 –World Wildlife Fund (Romania); 17 –Romanian Ornithological Society; 18 –University of Bucharest (other numbers according to S3 Table).

Fig 4 shows the three networks that were created–the entire network, the sub-network of planned actions and the sub-network of completed projects. Data on the networks’ characteristics are found in S2 Table. Data on nodes’ centrality in the networks are found in S3–S5 Tables.

The entire network of actors has a low density (6.4%) suggesting a loosely connected matrix. The average path length and diameter are both high (respectively 3.532 and 7) for such a spare network, which suggests that knowledge transfer and collaboration are difficult. The 57 actors are connected by 82 unique edges and 228 edges with duplicates. In terms of nodes, the mean degree is 3.579 (standard deviation = 2.802), mean betweenness is 58.719 (standard deviation = 163.590), and mean eigenvector is 0.018 (standard deviation = 0.017). The most connected actors are the National Authority for Waters (degree = 16; eigenvector = 0.069) and Romanian Ornithological Society (betweenness = 677.000). In terms of edges, the mean value per pair of nodes is 3.366 (standard deviation = 6.923). The strongest connections are between the National Authority for Waters and the Ministry of Environment, Waters and Forests (i.e., 38) and generally between the public authorities for water.

The sub-network of actors responsible for the planned actions has a low network density of 16.4% and a reduced average path length and diameter (respectively 1.694 and 2). The 22 actors are connected by 22 unique edges and 212 edges with duplicates. The mean degree of nodes is 3.455 (standard deviation = 3.203), mean betweenness 4.909 (standard deviation = 21.410), mean eigenvector = 0.045 (standard deviation = 0.038). The network is dominated by the National Authority for Water (degree = 16, normalized betweenness = 100.500, eigenvector = 0.162). The mean number of edges between two nodes is 6.947 (standard deviation = 10.358). Similar to the overall network, the strongest connection is between the National Authority for Waters and the Ministry of Environment, Waters and Forests (i.e., 38).

The sub-network of actors in completed projects has a low network density (9.60%) and a higher average path length and diameter (respectively 2.811 and 6). The 37 actors are connected by 60 unique edges and 16 edges with duplicates. The mean degree of nodes is 3.459 (standard deviation = 2.292), mean betweenness 24.514 (standard deviation = 65.387), and the mean eigenvector equals to 0.0270 (standard deviation = 0.028). The most connected actor is the Romanian Ornithological Society (degree = 11; betweenness = 273.000; eigenvector = 0.116). The mean number of edges between two nodes is 1.206 (standard deviation = 0.826). The strongest tie is between the Danube Delta Biosphere Reserve Administration and the Danube Delta Research and Development Institute (i.e., 6).

We found that the two sub-networks–of actors in planned actions versus completed projects–have differences and similarities. The sub-network of planned actions contains mainly public authorities for water management while the sub-network of completed projects has a larger variety of actors among which a single public authority for water. The sub-network of planned actions is smaller, with a lower average path length. The sub-network of completed projects has a higher number of actors that are less interconnected (i.e., more unique edges, lower number of edges per node). The sub-network of planned actions has a main powerful actor while the network of completed projects has several important actors. Both sub-networks have low density and low mean values of nodes’ centrality metrics. The two sub-networks have only two common actors, namely the River Basin Authority Prut-Bârlad and Danube Delta National Institute for Research and Development.

As a particularity of the sub-network of planned actions, actors collaborate on top-down hierarchical level. The river basin authorities work with the National Authority for Water and the Ministry of Environment, Waters and Forests. The river basin authorities have not worked with each other so far for river restoration.

As a particularity of the sub-network of completed projects, actors working on the Danube Delta have several edges with duplicates. All the other actors are connected by single edges; therefore, they collaborated in a single project. We also found that two completed projects were independent, actors worked only with each other, and therefore, these projects do not belong to the network.

### Holistic mapping of stakeholders

**Fig 5:**
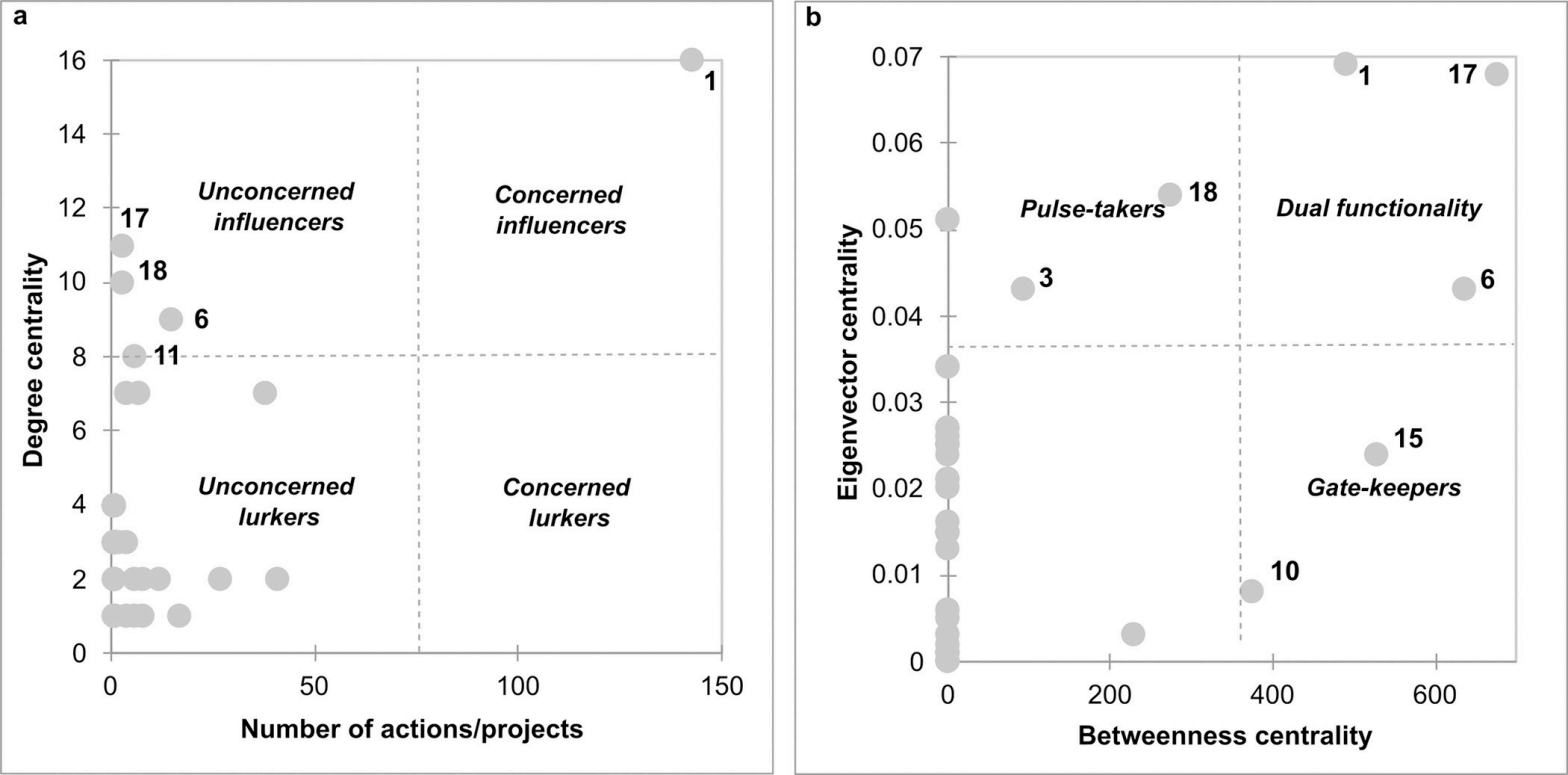
Holistic map of actors in river restoration in Romania: a) relation between the number of actions/projects per actor and degree of nodes in the network; b) relation between the betweenness and eigenvector of nodes in the network: 1 –National Authority of Waters; 3 –Ministry of Environment, Waters and Forests; 6 –River Basin Authority Prut-Barlad; 10 –Danube Delta Biosphere Reserve Authority; 11 –Danube Delta National Institution for Research and Development; 15 –World Wildlife Fund (Romania); 17 –Romanian Ornithological Society; 18 –University of Bucharest.

The number of projects per actor correlates with the actor’s degree (Spearman = 0.288; p-value = 0.036), which shows that the network has some important actors (Fig 5A). The network has a single concerned influencer–the National Authority for Water, with a great interest in river restoration and the highest degree in the network. The network has seven unconcerned influencers that do not express a particular interest in river restoration, but which have connections within the examined network. The unconcerned influencers are: the Romanian Ornithological Society, University of Bucharest, River Basin Authority Prut-Bârlad, and Danube Delta National Institute for Research and Development. All the other actors are unconcerned lurkers who have neither connections with other members in the network, nor do they express a particular interest in river restoration.

The eigenvector correlates with the betweenness of actors in the network (Spearman = 0.398, p-value = 0.003) showing the same actors are connected with both important nodes and disconnected/isolated ones (Fig 5B). The network has three actors with dual functionality (i.e., the River Basin Authority Prut-Bârlad, Romanian Ornithological Society, National Authority for Waters), two gate-keepers, and two pulse-takers. All the other actors play no particular role in the network.

## Discussion

So far, we identified the solutions and actors involved in river restoration in Romania, characterized the position of actors through social network analysis, and mapped the role of stakeholders through a holistic model. In what follows, we discuss the role of each central actor in the network.

The main actors of river restoration in Romania are: public authorities–the National Authority for Water, River Basin Authority Prut-Bârlad, Ministry of Environment, Waters and Forests, and Danube Delta Reserve Biosphere Administration; research–the Danube Delta National Institute for Research and Development and University of Bucharest; NGOs–the Romanian Ornithological Society and WWF Romania. In the overall network, the National Authority for Water is the most powerful actor in terms of degree, while the other actors act as bridges (i.e., high betweenness) between sub-networks of planned actions and completed projects.

The National Authority for Water has a central position in the network, because it is responsible for implementing the EU water and flood policy in Romania, together with the line ministry and river basin authorities. These prerogatives and divisions of responsibility are similar to other EU Member States. In effect, surveys applied in the EU Member States found that public authorities were considered the leading national actors in fulfilling the WFD aims [45, 46]. However, in Romania, except for River Basin Authority Prut-Bârlad, river basin authorities are grossly underrepresented. They are involved in several planned actions, but the corresponding node-level indicators have low values. Such an example is the one of the River Basin Authority Arges-Vedea; this river basin experiences the most severe pressure in Romania due to the presence within its bounds of the country capital, the city of Bucharest, and the priority given to flood risk defense and water supply in this area [47]. The underrepresentation of public institutions in Romania may also be a result of their low institutional capacity to initiate or participate in complex projects, such as understaffing, lack of proper training, financial constraints, or lack of capacity for linking local issues to EU water targets [48, 49]. Additionally, strong ties between certain authorities for water could show that actors are less likely to be exposed to new ideas [50].

The Ministry of Environment, Waters and Forests occupies a central position in the network compared to the other actors involved in river restoration in Romania. It is responsible for implementing actions related to opening floodplains and inundating floodplains. This is due to this ministry’s responsibility for flood defense and financial resources. In a study on the Lower Danube floodplain in Romania, Armas, Ionescu & Nenciu Posner [51] found that lower-income households expect some form of assistance not from the community, the church, or local authorities, but from the government. Based on this example, central authorities appear to have credibility at a local scale. Therefore, directly involving the Ministry of Environment, Waters and Forests in river governance may suit better, in most occasions, the local communities in Romania.

NGOs and researchers are willing to be successfully involved in environmental governance in Romania [22] in spite of the hierarchical, non-inclusive state system, which is still dominant [24]. The NGOs and researchers have a great potential to spread knowledge in the network (i.e., high betweenness) or to get maximum insight (i.e., high eigenvector). One positive example in this sense would be the Romanian Ornithological Society that has the ability to access both the central nodes and the wider community in the context of a low number of completed projects (3). We also highlight the proficiency of the University of Bucharest to obtain funds, work with various partners and hypothetically bring new ideas in the network. These examples confirm that river restoration is not an exclusive interest of public authorities in Romania, but a polycentric system of managing resources [52, 53] in which scientists bring their know-how [54] and NGOs provide democratic legitimacy to governance processes [55].

For the case study of Romania, we consider that a recipe for success is the collaboration between public authorities with field experience and researchers [56], because most of the projects that got financed relied on this partnership as forms of co-management [57, 58]. The state agencies support and finance the restoration of the rural river channels (e.g., strictly protected areas) avoiding payback measures and relocation [6]. Research organizations come with their technical expertise, contribute to writing initial projects and have the proper training to apply for calls for projects [22]. The most obvious example in Romania is the strong relationship between the Danube Delta Biosphere Reserve Authority and the Danube Delta National Institute for Research and Development with six common projects completed in the Danube Delta, which is the region with the highest status of environment protection in Romania (i.e., UNESCO). These strong collaborations also show a high level of trust between the partners that is very important for the polycentric governance [59].

Concerning the scale, international partners always work with public authorities and researchers. On the contrary, local partners are missing from this successful partnership. In Romania, restoration solutions are mostly perceived as technical projects, with no interest in local traditional knowledge [9]. The presence of international partners that exclude local partners suggests a dominant top-down approach in river restoration in Romania, as well the need for technical expertise. For instance, Bursan & Mitroi-Tisseyre [60] showed that local villagers were against restoration practices in a certain site in the Danube Delta and proposed their own vision for river restoration. Ignoring the local ecological knowledge in this case is probably due to the status of protected areas that requires focus on conserving biodiversity. Further transdisciplinary studies such as those in anthropology could place the policy makers in a better position to consider the local community in future planning of river restoration.

Concerning the actors of river restoration, we found similarities, as well as particularities when compared to other EU Member States. Among them, we noticed a multiplicity of actors and polycentric governance in river restoration similar to case studies in France [61, 62], Netherlands [21] or Germany [63]. We underlined the dominant and controlling role of public authorities that ensure the connectedness of non-governmental actors similar to a case study in Netherlands [21]. These public authorities exert their responsibilities at regional scale, which is not unexpected given the fact that traditional decision-making is mostly controlled by the state, with the consultation of non-state actors [64]. Unlike other case studies that inventoried numerous, small actors at a local scale [62], a major part of river restoration projects in Romania includes actors that operate at the national scale. Additionally, the presence of international actors is somewhat contrary to the role of restoration as a social process [65]. We confirmed that flood defense remains an enduring priority in countries exposed to flood risk [18] through the presence of the Ministry of Environment, Waters and Forests in all actions related to opening floodplains in Romania. This type of top-down approach was also shown in dyke relocation actions thus flood risk management in Germany by Zingraff-Hamed and collaborators [63] similar to the case study of Romania.

In terms of limitations of our research, we only presented actors that apply for funding for river restoration. Behind the actors responsible for planned actions and partners in ongoing/completed projects, other actors can be employed such as technical experts and constructors. These actors are not necessarily interested in river restoration, but are certainly responsible for achieving effective results. Further analysis based on a project-by-project approach could deepen the understanding of the role of each stakeholder in a river restoration solution.

## Conclusions

Our study highlighted the most central actors in river restoration in Romania in both planned actions and completed projects: national- and regional-level stakeholders such as authorities for water and environment, universities and NGOs. We found that the National Authority for Water creates the policy of river restoration through the high number of planned actions and numerous partners especially in the area of water. Yet, other actors such as NGOs and researchers appear to be interested in river restoration as well. We noticed that international partners are preferred to local partners. These findings show that the technical expertise of actors is important in river restoration in Romania to the expense of local indigenous knowledge and societal benefits.

Romania’s ‘governance style’ in river restoration is an example of how the EU requirements are translated by an EU Member State. Still, the ‘governance style’ depends on numerous factors that surpass in importance the EU policy, among which we note the previous organization of the water and environment resources, socio-cultural differences [6], extreme hydroclimatic events [30], and a limited capacity to design and submit projects to gain funds [22]. As recommendation for governing river restoration in Romania, we urge the creation of a national programme focused on river restoration with call for projects coordinated by the National Authority for Water, open to river basin authorities (grossly underrepresented so far) in agreement with stakeholders from various sectors, beyond the water domain [66]. Additionally, a more effective collaboration between actors in various projects could improve communication and sharing their know-how towards diversifying river restoration goals at national scale.

Our study is a contribution to a better understanding of the governance of river restoration through an objective approach. Further comparative studies (across geographic regions, governance scales and/or bottlenecks) can eventually help to ascertain specific findings, that may potentially provide the basis for more insightful recommendations for EU river restoration policy [66]. One such recommendation, much needed in the river restoration field, would be the access to funds and well-trained teams able to manage rivers from an ecological perspective. Additionally, our study is an illustrative example of the involvement of research organizations in completing river restoration that could be further cited as an example of good practice.

## Supporting information

S1 Table Actors in river restoration in Romania related by edges. (DOCX)

S2 Table Characteristics of the network of actors in river restoration in Romania and of comprised sub-networks. (DOCX)

S3 Table Actors in river restoration in Romania–entire network. (DOCX)

S4 Table Actors in river restoration in Romania–sub-network of actors in planned actions. (DOCX)

S5 Table Actors in river restoration in Romania–sub-network of actors in completed actions. (DOCX)
