# Supplementary material for: Assessing the role of actors in river restoration: A network perspective
Source: PLoS One. 2024 Apr 16;19(4):e0297745. doi: 10.1371/journal.pone.0297745 (PMC11020697; doi:10.1371/journal.pone.0297745)
Supplement: S1 Table — (DOCX) [file pone.0297745.s001.docx]

**Table S1. Actors in river restoration in Romania related by edges.**

| **Network** | **Actor** | **Actor** | **Number of edges** |
| --- | --- | --- | --- |
| **Sub-network**  **completed projects** | River Basin Authority Prut-Bârlad | University of Bucharest | 1 |
|  | River Basin Authority Prut-Bârlad | Romanian Ornithological Society | 1 |
|  | River Basin Authority Prut-Bârlad | Forestry Agency Galați | 1 |
|  | River Basin Authority Prut-Bârlad | Environment Protection Agency Galați | 1 |
|  | University of Bucharest | Romanian Ornithological Society | 1 |
|  | University of Bucharest | Forestry Agency Galați | 1 |
|  | University of Bucharest | Environment Protection Agency Galați | 1 |
|  | Romanian Ornithological Society | Forestry Agency Galați | 1 |
|  | Romanian Ornithological Society | Environment Protection Agency Galați | 1 |
|  | Forestry Agency Galați | Environment Protection Agency Galați | 1 |
|  | Danube Delta National Institution for Research and Development | Forestry Agency Tulcea | 1 |
|  | World Wildlife Fund (Romania) | Lafarge Company | 1 |
|  | Danube Delta National Institution for Research and Development | Danube Delta Biosphere Reserve Authority | 6 |
|  | River Basin Authority Prut-Bârlad | Institute for Inland Water Management RIZA | 1 |
|  | River Basin Authority Prut-Bârlad | Regional Water Board Hunze&Aa's | 1 |
|  | River Basin Authority Prut-Bârlad | Het Drentse Landschap (Netherlands) | 1 |
|  | Institute for Inland Water Management RIZA (Netherlands) | Regional Water Board Hunze&Aa's (Netherlands) | 1 |
|  | Institute for Inland Water Management RIZA (Netherlands) | Het Drentse Landschap (Netherlands) | 1 |
|  | Regional Water Board Hunze&Aa's (Netherlands) | Het Drentse Landschap (Netherlands) | 1 |
|  | Forestry Agency Brăila | University of Bucharest | 1 |
|  | Comana Natural Park Authority | Environment Protection Agency Giurgiu | 1 |
|  | Comana Natural Park Authority | County Council Giurgiu | 1 |
|  | Comana Natural Park Authority | Local Council Comana | 1 |
|  | Environment Protection Agency Giurgiu | County Council Giurgiu | 1 |
|  | Environment Protection Agency Giurgiu | Local Council Comana | 1 |
|  | County Council Giurgiu | Local Council Comana | 1 |
|  | World Wildlife Fund (Romania) | Romanian Ornithological Society | 1 |
|  | World Wildlife Fund (Romania) | Environment Protection Agency Olt | 1 |
|  | World Wildlife Fund (Romania) | Environment Protection Agency Teleorman | 1 |
|  | Romanian Ornithological Society | Environment Protection Agency Olt | 1 |
|  | Romanian Ornithological Society | Environment Protection Agency Teleorman | 1 |
|  | Environment Protection Agency Olt | Environment Protection Agency Teleorman | 1 |
|  | Environment Protection Agency Gorj | University of Bucharest | 1 |
|  | Environment Protection Agency Gorj | Invisile Nature Consultancy | 1 |
|  | University of Bucharest | Invisile Nature Consultancy | 1 |
|  | Local Council Mahmudia | World Wildlife Fund (Romania) | 1 |
|  | Local Council Mahmudia | Danube Delta Biosphere Reserve Authority | 1 |
|  | World Wildlife Fund (Romania) | Danube Delta Biosphere Reserve Authority | 1 |
|  | World Wildlife Fund (Romania) | Coca Cola Company (Romania) | 1 |
|  | Danube Delta National Institution for Research and Development | Directorate for Public Works and Water Management (Netherlands) | 2 |
|  | Danube Delta National Institution for Research and Development | World Wildlife Fund (Auen Institut, Rastatt, Germany) | 4 |
|  | Directorate for Public Works and Water Management (Netherlands) | Danube Delta Biosphere Reserve Authority | 2 |
|  | World Wildlife Fund (Auen Institut, Rastatt, Germany) | Danube Delta Biosphere Reserve Authority | 4 |
|  | Local Council Tulcea | Danube Delta Biosphere Reserve Authority | 1 |
|  | National Agency for Land Improvement | Danube Delta Biosphere Reserve Authority | 1 |
|  | Local Council Tulcea | National Agency for Land Improvement | 1 |
|  | DACROM Company | National Institution for Research and Development for Forestry | 1 |
|  | University of Bucharest | Natural History Museum Grigore Natural History Museum Grigore Antipa | 1 |
|  | University of Bucharest | Iron Gates Natural Park Administration | 1 |
|  | University of Bucharest | Environment Protection Agency Caraș Severin | 1 |
|  | Natural History Museum Grigore Natural History Museum Grigore Antipa | Iron Gates Natural Park Administration | 1 |
|  | Natural History Museum Grigore Natural History Museum Grigore Antipa | Environment Protection Agency Caraș Severin | 1 |
|  | Iron Gates Natural Park Administration | Environment Protection Agency Caraș Severin | 1 |
|  | Environment Protection Agency Cluj | University of Cluj | 1 |
|  | Environment Protection Agency Cluj | Romanian Ornithological Society | 1 |
|  | Environment Protection Agency Cluj | The Romanian Academy | 1 |
|  | Environment Protection Agency Cluj | National Forestry Agency | 1 |
|  | University of Cluj | Romanian Ornithological Society | 1 |
|  | University of Cluj | The Romanian Academy | 1 |
|  | University of Cluj | National Forest Administration | 1 |
|  | Romanian Ornithological Society | The Romanian Academy | 1 |
|  | Romanian Ornithological Society | National Forestry Agency | 1 |
|  | The Romanian Academy | National Forestry Agency | 1 |
| **Sub-network**  **of planned actions** | Ministry of Environment, Waters and Forests | National Administration Romanian Waters | 38 |
|  | Ministry of Environment, Waters and Forests | River Basin Authority Buzău-Ialomița | 5 |
|  | National Administration Romanian Waters | River Basin Authority Buzău-Ialomița | 6 |
|  | Ministry of Environment, Waters and Forests | River Basin Authority Siret | 3 |
|  | National Administration Romanian Waters | River Basin Authority Siret | 8 |
|  | Ministry of Environment, Waters and Forests | River Basin Authority Prut-Bârlad | 15 |
|  | National Administration Romanian Waters | River Basin Authority Prut-Bârlad | 15 |
|  | National Administration Romanian Waters | River Basin Authority Argeș-Vedea | 1 |
|  | Ministry of Environment, Waters and Forests | River Basin Authority Jiu | 11 |
|  | National Administration Romanian Waters | River Basin Authority Jiu | 11 |
|  | National Administration Romanian Waters | River Basin Authority Crișuri | 27 |
|  | National Administration Romanian Waters | River Basin Authority Mureș | 8 |
|  | Ministry of Environment, Waters and Forests | River Basin Authority Crișuri | 1 |
|  | National Administration Romanian Waters | River Basin Authority Someș-Tisza | 36 |
|  | Ministry of Environment, Waters and Forests | River Basin Authority Someș-Tisza | 33 |
|  | National Administration Romanian Waters | River Basin Authority Banat | 6 |
|  | National Administration Romanian Waters | River Basin Authority Olt | 3 |
|  | National Administration Romanian Waters | River Basin Authority Dobrogea-Litoral | 17 |
|  | National Administration Romanian Waters | University of Natural Resources and Life Science Vienna | 1 |
|  | National Administration Romanian Waters | Danube River Basin Directorate | 1 |
|  | National Administration Romanian Waters | Croatian Waters - Legal entity for water management | 1 |
|  | National Administration Romanian Waters | Morava River Basin Authority | 1 |
|  | Danube Delta National Institution for Research and Development | International Commission for the Protection of the Danube River | 1 |
|  | Danube Delta National Institution for Research and Development | CDM Smith | 1 |
|  | Danube Delta National Institution for Research and Development | Jaroslav Černi Institute (JCI) | 1 |
|  | Danube Delta National Institution for Research and Development | Norwegian Institute for Nature Research | 1 |
