# Supplementary material for: Assessing the role of actors in river restoration: A network perspective
Source: PLoS One. 2024 Apr 16;19(4):e0297745. doi: 10.1371/journal.pone.0297745 (PMC11020697; doi:10.1371/journal.pone.0297745)
Supplement: S2 Table — (DOCX) [file pone.0297745.s002.docx]

**Table S2. Characteristics of the network of actors in river restoration in Romania and of comprised sub-networks.**

| **Measure** | **Metrics** | | **Entire network** | **Sub-network of**  **planned actions** | **Sub-network of**  **completed projects** |
| --- | --- | --- | --- | --- | --- |
| Network-level | Density | | 6.4 | 16.4 | 9.60 |
|  | Average path length | | 3.532 | 1.694 | 2.811 |
|  | Diameter | | 7 | 2 | 6 |
|  | Edges | Total | 298 | 222 | 76 |
|  |  | Unique edges | 82 | 22 | 60 |
|  |  | Edges with duplicates | 228 | 212 | 16 |
| Network-level | Total number | | 57 | 22 | 37 |
|  | Degree | Mean | 3.579 | 3.455 | 3.459 |
|  |  | St. dev. | 2.802 | 3.203 | 2.292 |
|  | Betweenness | Mean | 58.719 | 4.909 | 24.514 |
|  |  | St. dev. | 163.590 | 21.410 | 65.387 |
|  | Eigenvector | Mean | 0.018 | 0.045 | 0.027 |
|  |  | St. dev. | 0.017 | 0.038 | 0.028 |
|  | Edges | Mean | 3.366 | 6.947 | 1.206 |
|  |  | St. dev | 6.923 | 10.358 | 0.826 |
|  | Sector | Public authorities | 37 | 17 | 21 |
|  |  | Research | 12 | 4 | 9 |
|  |  | NGO | 4 | 0 | 4 |
|  |  | Private company | 4 | 1 | 3 |
|  | Scale | International | 14 | 8 | 6 |
|  |  | National | 16 | 3 | 14 |
|  |  | Regional | 23 | 11 | 13 |
|  |  | Local | 4 | 0 | 4 |
