# Supplementary material for: Assessing the role of actors in river restoration: A network perspective
Source: PLoS One. 2024 Apr 16;19(4):e0297745. doi: 10.1371/journal.pone.0297745 (PMC11020697; doi:10.1371/journal.pone.0297745)
Supplement: S3 Table — (DOCX) [file pone.0297745.s003.docx]

**Table S3. Actors in river restoration in Romania – entire network.**

| **No** | **Actor** | **Scale** | **Type** | **Number of actions/projects** | **Degree centrality** | **Betweenness centrality** | **Eigenvector centrality** |
| --- | --- | --- | --- | --- | --- | --- | --- |
| 1 | National Administration Romanian Waters | National | Public authority | 143 | 16 | 491.500 | 0.069 |
| 2 | River Basin Authority Someș-Tisza | Basin | Public authority | 41 | 2 | 0.000 | 0.025 |
| 3 | Ministry of Environment, Waters and Forests | National | Public authority | 38 | 7 | 92.500 | 0.043 |
| 4 | River Basin Authority Crișuri | Basin | Public authority | 27 | 2 | 0.000 | 0.025 |
| 5 | River Basin Authority Dobrogea-Litoral | Basin | Public authority | 17 | 1 | 0.000 | 0.015 |
| 6 | River Basin Authority Prut-Bârlad | Basin | Public authority | 15 | 9 | 637.000 | 0.043 |
| 7 | River Basin Authority Jiu | Basin | Public authority | 12 | 2 | 0.000 | 0.025 |
| 8 | River Basin Authority Mureș | Basin | Public authority | 8 | 1 | 0.000 | 0.015 |
| 9 | River Basin Authority Siret | Basin | Public authority | 8 | 2 | 0.000 | 0.025 |
| 10 | Danube Delta Biosphere Reserve Authority | Regional | Public authority | 7 | 7 | 376.000 | 0.008 |
| 11 | Danube Delta National Institution for Research and Development | National | Research organization | 6 | 8 | 229.000 | 0.003 |
| 12 | River Basin Authority Banat | Basin | Public authority | 6 | 1 | 0.000 | 0.015 |
| 13 | River Basin Authority Buzău-Ialomița | Basin | Public authority | 6 | 2 | 0.000 | 0.025 |
| 14 | River Basin Authority Olt | Basin | Public authority | 4 | 1 | 0.000 | 0.015 |
| 15 | World Wildlife Fund (Romania) | National | NGO | 4 | 7 | 529.000 | 0.024 |
| 16 | World Wildlife Fund (Auen Institut, Rastatt, Germany) | International | NGO | 4 | 3 | 0.000 | 0.003 |
| 17 | [Romanian Ornithological Society](https://www.birdlife.org/partners/romania-romanian-ornithological-society-sor-birdlife-romania/) | National | NGO | 3 | 11 | 677.000 | 0.068 |
| 18 | University of Bucharest | National | Research organization | 3 | 10 | 275.000 | 0.054 |
| 19 | Directorate for Public Works and Water Management (Netherlands) | International | Public authority | 2 | 3 | 0.000 | 0.003 |
| 20 | CDM Smith | International | Private company | 1 | 4 | 0.000 | 0.001 |
| 21 | Coca Cola Foundation USA | International | Private company | 1 | 1 | 0.000 | 0.005 |
| 22 | Comana Natural Park Authority | Local | Public authority | 1 | 3 | 0.000 | 0.000 |
| 23 | County Council Giurgiu | County | Public authority | 1 | 3 | 0.000 | 0.000 |
| 24 | Croatian Waters - Legal entity for water management | International | Public authority | 1 | 4 | 0.000 | 0.027 |
| 25 | DACROM Company | National | Private company | 1 | 1 | 0.000 | 0.000 |
| 26 | Danube River Basin Directorate | International | Public authority | 1 | 4 | 0.000 | 0.027 |
| 27 | Environment Protection Agency Caraș-Severin | County | Public authority | 1 | 3 | 0.000 | 0.021 |
| 28 | Environment Protection Agency Cluj | County | Public authority | 1 | 4 | 0.000 | 0.034 |
| 29 | Environment Protection Agency Galați | Regional | Public authority | 1 | 4 | 0.000 | 0.051 |
| 30 | Environment Protection Agency Giurgiu | County | Public authority | 1 | 3 | 0.000 | 0.000 |
| 31 | Environment Protection Agency Gorj | County | Public authority | 1 | 2 | 0.000 | 0.016 |
| 32 | Environment Protection Agency Olt | County | Public authority | 1 | 3 | 0.000 | 0.020 |
| 33 | Environment Protection Agency Teleorman | County | Public authority | 1 | 3 | 0.000 | 0.020 |
| 34 | Forestry Agency Brăila | County | Public authority | 1 | 1 | 0.000 | 0.013 |
| 35 | Forestry Agency Galați | County | Public authority | 1 | 4 | 0.000 | 0.051 |
| 36 | Forestry Agency Tulcea | County | Public authority | 1 | 1 | 0.000 | 0.001 |
| 37 | Het Drentse Landschap (Netherlands) | International | NGO | 1 | 3 | 0.000 | 0.026 |
| 38 | Institute for Inland Water Management RIZA (Netherlands) | International | Research organization | 1 | 3 | 0.000 | 0.026 |
| 39 | International Commission for the Protection of the Danube River | International | Public authority | 1 | 4 | 0.000 | 0.001 |
| 40 | Invisile Nature Consultancy | National | Research organization | 1 | 2 | 0.000 | 0.016 |
| 41 | Iron Gates Natural Park Administration | Regional | Public authority | 1 | 3 | 0.000 | 0.021 |
| 42 | Jaroslav Černi Institute | International | Research organization | 1 | 4 | 0.000 | 0.001 |
| 43 | Lafarge Company | National | Private company | 1 | 1 | 0.000 | 0.005 |
| 44 | Local Council Comana | Local | Public authority | 1 | 3 | 0.000 | 0.000 |
| 45 | Local Council Mahmudia | Local | Public authority | 1 | 2 | 0.000 | 0.006 |
| 46 | Local Council Tulcea | Local | Public authority | 1 | 2 | 0.000 | 0.002 |
| 47 | Morava River Basin Authority | International | Public authority | 1 | 4 | 0.000 | 0.027 |
| 48 | National Agency for Land Improvement | National | Public authority | 1 | 2 | 0.000 | 0.002 |
| 49 | National Institution for Research and Development for Forestry | National | Research organization | 1 | 1 | 0.000 | 0.000 |
| 50 | National Forestry Administration | National | Public authority | 1 | 4 | 0.000 | 0.006 |
| 51 | Natural History Museum Grigore Antipa | National | Research organization | 1 | 3 | 0.000 | 0.021 |
| 52 | Norwegian Institute for Nature Research | International | Research organization | 1 | 4 | 0.000 | 0.001 |
| 53 | Regional Water Board Hunze&Aa's (Netherlands) | International | Public authority | 1 | 3 | 0.000 | 0.026 |
| 54 | River Basin Authority Argeș-Vedea | Basin | Public authority | 1 | 1 | 0.000 | 0.015 |
| 55 | The Romanian Academy | National | Research organization | 1 | 4 | 0.000 | 0.034 |
| 56 | University of Cluj | National | Research organization | 1 | 4 | 0.000 | 0.034 |
| 57 | University of Natural Resources and Life Science Vienna | International | Research organization | 1 | 4 | 0.000 | 0.024 |
