# Supplementary material for: Assessing the role of actors in river restoration: A network perspective
Source: PLoS One. 2024 Apr 16;19(4):e0297745. doi: 10.1371/journal.pone.0297745 (PMC11020697; doi:10.1371/journal.pone.0297745)
Supplement: S4 Table — (DOCX) [file pone.0297745.s004.docx]

**Table S4. Actors in river restoration in Romania – sub-network of actors in planned actions.**

| **Actor** | **Degree centrality** | **Betweenness centrality** | **Eigenvector centrality** |
| --- | --- | --- | --- |
| National Administration Romanian Waters | 16 | 100.500 | 0.162 |
| Ministry of Environment, Waters and Forests | 7 | 7.500 | 0.088 |
| Danube Delta National Institution for Research and Development | 4 | 0.000 | 0.000 |
| University of Natural Resources and Life Science Vienna | 4 | 0.000 | 0.075 |
| Danube River Basin Directorate | 4 | 0.000 | 0.075 |
| Croatian Waters - Legal entity for water management | 4 | 0.000 | 0.075 |
| Morava River Basin Authority | 4 | 0.000 | 0.075 |
| International Commission for the Protection of the Danube River | 4 | 0.000 | 0.000 |
| CDM Smith | 4 | 0.000 | 0.000 |
| Jaroslav Černi Institute | 4 | 0.000 | 0.000 |
| Norwegian Institute for Nature Research | 4 | 0.000 | 0.000 |
| River Basin Authority Buzău-Ialomița | 2 | 0.000 | 0.049 |
| River Basin Authority Crișuri | 2 | 0.000 | 0.049 |
| River Basin Authority Someș-Tisza | 2 | 0.000 | 0.049 |
| River Basin Authority Siret | 2 | 0.000 | 0.049 |
| River Basin Authority Prut-Bârlad | 2 | 0.000 | 0.049 |
| River Basin Authority Jiu | 2 | 0.000 | 0.049 |
| River Basin Authority Argeș-Vedea | 1 | 0.000 | 0.031 |
| River Basin Authority Mureș | 1 | 0.000 | 0.031 |
| River Basin Authority Banat | 1 | 0.000 | 0.031 |
| River Basin Authority Olt | 1 | 0.000 | 0.031 |
| River Basin Authority Dobrogea-Litoral | 1 | 0.000 | 0.031 |
