# Supplementary material for: Assessing the role of actors in river restoration: A network perspective
Source: PLoS One. 2024 Apr 16;19(4):e0297745. doi: 10.1371/journal.pone.0297745 (PMC11020697; doi:10.1371/journal.pone.0297745)
Supplement: S5 Table — (DOCX) [file pone.0297745.s005.docx]

**Table S5. Actors in river restoration in Romania – sub-network of actors in completed actions.**

| **Actor** | **Degree centrality** | **Betweenness centrality** | **Eigenvector centrality** |
| --- | --- | --- | --- |
| [Romanian Ornithological Society](https://www.birdlife.org/partners/romania-romanian-ornithological-society-sor-birdlife-romania/) | 11 | 273.000 | 0.116 |
| University of Bucharest | 10 | 155.000 | 0.087 |
| World Wildlife Fund (Romania) | 7 | 217.000 | 0.044 |
| Danube Delta Biosphere Reserve Authority | 7 | 152.000 | 0.014 |
| River Basin Authority Prut-Bârlad | 7 | 81.000 | 0.076 |
| Danube Delta National Institution for Research and Development | 4 | 29.000 | 0.004 |
| Forestry Agency Galați | 4 | 0.000 | 0.065 |
| Environment Protection Agency Galați | 4 | 0.000 | 0.065 |
| Environment Protection Agency Cluj | 4 | 0.000 | 0.051 |
| University of Cluj | 4 | 0.000 | 0.051 |
| The Romanian Academy | 4 | 0.000 | 0.051 |
| National Forest Administration | 4 | 0.000 | 0.051 |
| World Wildlife Fund (Auen Institut, Rastatt, Germany) | 3 | 0.000 | 0.004 |
| Directorate for Public Works and Water Management (Netherlands) | 3 | 0.000 | 0.004 |
| Institute for Inland Water Management RIZA | 3 | 0.000 | 0.023 |
| Regional Water Board Hunze&Aa's | 3 | 0.000 | 0.023 |
| Het Drentse Landschap | 3 | 0.000 | 0.023 |
| Comana Natural Park Authority | 3 | 0.000 | 0.000 |
| Environment Protection Agency Giurgiu | 3 | 0.000 | 0.000 |
| County Council Giurgiu | 3 | 0.000 | 0.000 |
| Local Council Comana | 3 | 0.000 | 0.000 |
| Environment Protection Agency Olt | 3 | 0.000 | 0.037 |
| Environment Protection Agency Teleorman | 3 | 0.000 | 0.037 |
| Natural History Museum Grigore Antipa | 3 | 0.000 | 0.026 |
| Environment Protection Agency Caraș-Severin | 3 | 0.000 | 0.026 |
| Iron Gates Natural Park Administration | 3 | 0.000 | 0.026 |
| Environment Protection Agency Gorj | 2 | 0.000 | 0.020 |
| Invisile Nature Consultancy | 2 | 0.000 | 0.020 |
| Local Council Mahmudia | 2 | 0.000 | 0.011 |
| Local Council Tulcea | 2 | 0.000 | 0.003 |
| National Agency for Land Improvement | 2 | 0.000 | 0.003 |
| Forestry Agency Tulcea | 1 | 0.000 | 0.001 |
| Lafarge Company | 1 | 0.000 | 0.008 |
| Forestry Agency Brăila | 1 | 0.000 | 0.016 |
| Coca Cola Foundation USA | 1 | 0.000 | 0.008 |
| National Institution for Research and Development for Forestry | 1 | 0.000 | 0.000 |
| DACROM Company | 1 | 0.000 | 0.000 |
